# Anomalous switching in Nb/Ru/Sr_2_RuO_4_ topological junctions by chiral domain wall motion

**DOI:** 10.1038/srep02480

**Published:** 2013-08-21

**Authors:** M. S. Anwar, Taketomo Nakamura, S. Yonezawa, M. Yakabe, R. Ishiguro, H. Takayanagi, Y. Maeno

**Affiliations:** 1 Department of Physics, Kyoto University, Kyoto 606-8502, Japan; 2 Institute for Solid State Physics, the University of Tokyo, Kashiwa 277-8581, Japan; 3 Department of Applied Physics, Faculty of Science, Tokyo University of Science, Tokyo 162-8601, Japan; 4 International Center for Materials Nanoarchitectonics (MANA), National Institute for Materials Science (NIMS), Tsukuba 305-0044, Japan

## Abstract

A spontaneous symmetry breaking in a system often results in domain wall formation. The motion of such domain walls is utilized to realize novel devices like racetrack-memories, in which moving ferromagnetic domain walls store and carry information. Superconductors breaking time reversal symmetry can also form domains with degenerate chirality of their superconducting order parameter. Sr_2_RuO_4_ is the leading candidate of a chiral *p*-wave superconductor, expected to be accompanied by chiral domain structure. Here, we present that Nb/Ru/Sr_2_RuO_4_ topological superconducting-junctions, with which the phase winding of order parameter can be effectively probed by making use of real-space topology, exhibit unusual switching between higher and lower critical current states. This switching is well explained by chiral-domain-wall dynamics. The switching can be partly controlled by external parameters such as temperature, magnetic field and current. These results open up a possibility to utilize the superconducting chiral domain wall motion for future novel superconducting devices.

Since the discovery of superconductivity in Sr_2_RuO_4_ (SRO)^1^, various experiments^2^,^3^,^4^,^5^,^6^,^7^ reveal that the pairing state of SRO is of chiral *p*-wave spin-triplet with broken time reversal symmetry^8^,^9^, analogous to the A-phase of superfluid ^3^He^10^,^11^. However, the issue of chiral *p*-wave nature of SRO still remains controversial since some of the predicted behavior such as chiral edge current has not been observed^9^. Thus establishment of novel behavior specific to chiral *p*-wave superconductivity is much desirable. Recently, SRO is considered as one of the most promising materials for exploring topological superconducting phenomena originating from its orbital phase winding^9^. Because of nontrivial topological aspect of its superconducting order parameter, gapless chiral edge states consisting of Majorana quasiparticles (whose antiparticles are their own particles) are believed to emerge at its boundaries^12^,^13^,^14^,^15^.

Chiral *p*-wave superconductivity exhibits two-fold degeneracy corresponding to clockwise or counterclockwise winding of the superconducting phase. This degeneracy sets up two kinds of chiral domains separated by a chiral domain wall (chiral-DW)^16^. To date, there is no direct observation of the chiral-DW^17^. However, the existence of the chiral-DW has been strongly suggested by transport studies of SRO-based junctions^2^,^18^. Further accumulation of evidence of the chiral-DW and investigations of possible influences of chiral-DW dynamics on transport properties are important because the chiral-DW can be utilized for novel superconducting devices as in the case of the ferromagnetic-DW for racetrack memory devices^19^.

A “topological junction” consists of a superconductor surrounded by another in such a way that the difference in phase winding dictates the junction behavior^20^,^21^. The characteristics of a topological junction with a chiral *p*-wave superconductor should be very sensitive to the chiral domain configuration. The SRO-Ru eutectic system^22^ provides naturally existing topological junctions, once *s*-wave superconductivity is induced into Ru inclusions surrounded by SRO. Indeed, junctions fabricated using Pb as an *s*-wave superconductor deposited over many Ru-inclusions^20^,^21^ exhibit peculiar temperature dependence of critical current *I*_c_ attributable to topological phase competition between the *s*-wave and *p*-wave superconductivity. Since previous devices containing many Ru junctions probe only averaged effects, it is much desirable to fabricate a device with a single junction to investigate the order parameter structure more sensitively, including the effect of chiral domains.

## Results

**Figure 1:**
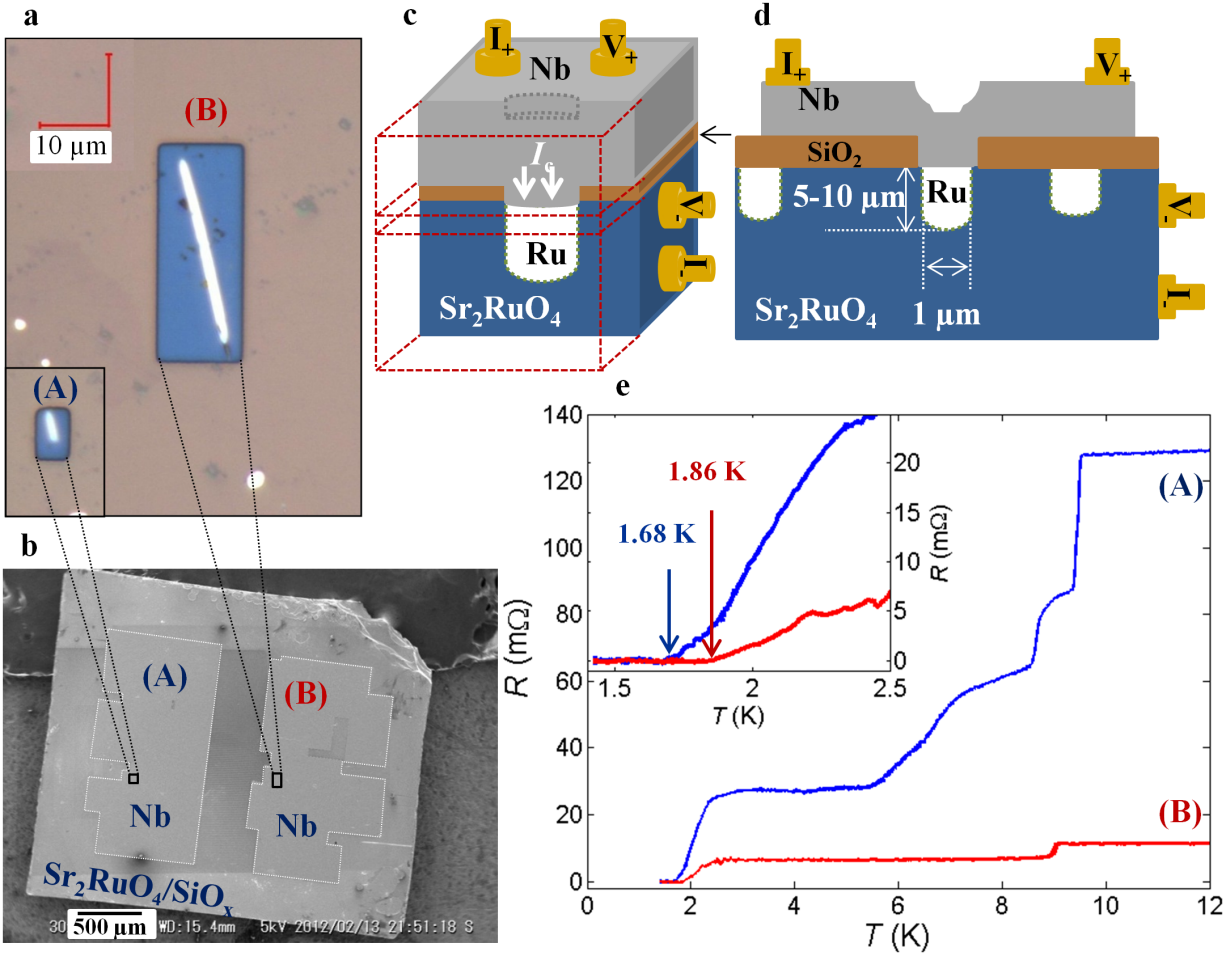
Topological junction devices and the resistance versus temperature. (a), Optical microscope image of the Nb contact area with SRO over a single Ru inclusion of cross sectional area 1 × 5 μm^2^ for junction A (shown in the inset) and 1 × 14 μm^2^ for Junction B. The elongated bright part in the blue box (where SiO_x _layer is etched out) is the Ru inclusion and blue indicates SRO. (b), Scanning Electron Microscope (SEM) image of the Nb/Ru/SRO device with two junctions A and B. White dotted lines show the boundary of Nb electrodes and black rectangular areas indicate the positions of the junctions. Note that the contrast between Ru and SRO is clear under optical microscope but not under SEM. That is the reason we used optical microscope for the zoomed-in image. (c), 3D schematic view of a junction. (d), Schematic cross section of the junction, fabricated by depositing ~1 μm thick Nb electrode after depositing ~300 nm thick SiO_x_ layer with an open window over a single Ru inclusion. (e), Resistance versus temperature for both Junctions A and B. The normal junction resistance *R*_N_ is 128 mΩ for the junction A (blue curve) and 11.5 mΩ for the junction B (red curve). The different *R*_N_ values are attributed to different interface transparency as well as cross sectional area. The drop in the resistance at around 9 K corresponds to superconducting transition of Nb (*T*_c_ ≈ 9.5 K) and the drop to zero resistance starts at 2.8 K because of proximity effect with 3-K phase. There are additional drops for the junction A, reflecting gradual development of proximity into Ru metal. The inset is an enlargement showing that the zero junction resistance persists to temperatures substantially above *T*_c_bulk_ = 1.42 K of SRO. The bulk resistance of SRO is negligible compared with the junction resistance.

**Figure 2:**
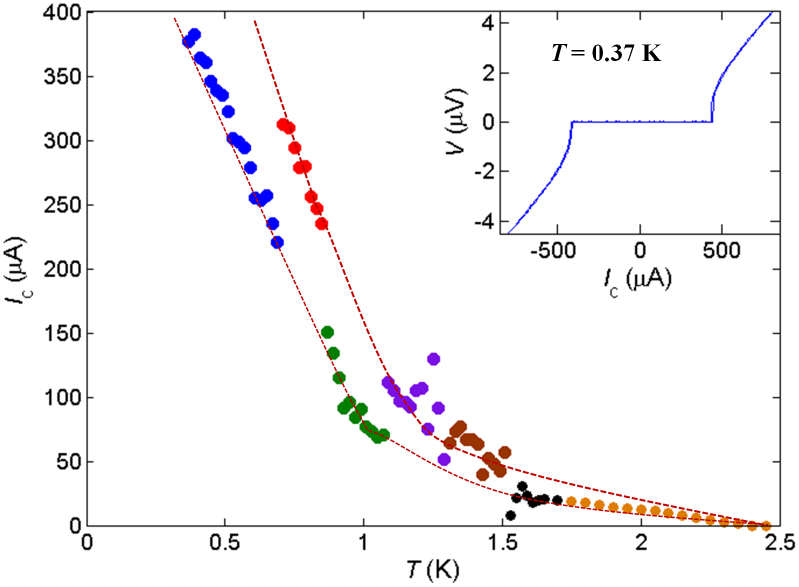
Temperature dependent critical current of the topological junctions. Critical current *I*_c_ as a function of temperature *T* at zero field for Junctions B. During the measurements the temperature was raised above 3 K several times due to the liquid ^3^He hold time of our refrigerator. To continue our measurements we cooled it down again. In this way the *I*_c_(*T*) data were accumulated with various cooling cycles: different colors represent cooling cycles. There are sharp *I*_c_ jumps that start with every subsequent cooling cycle, indicating two different *I*_c_ braches. The dotted red curves illustrate two different *I*_c_ branches. Inset shows a current-voltage (*I*-*V*) curve at 0.37 K for Junction B. It shows a clear zero voltage supercurrent up to ~450 μA in the most stable state.

We fabricate SRO-Ru based micron-sized junctions utilizing only one Ru inclusion shown in Figs. 1a–d. Figure 1e presents the junction resistance versus temperature. Junction A (Junction B) exhibits the first transition at 9.5 K (9 K) corresponding to the superconducting transition temperature *T*_c_ of Nb. The final transition starting at around 2.8 K (for both junctions) leads to zero junction resistance at *T_c,A_* = 1.68 K (*T_c,B_* = 1.68 K) (inset of Fig. 1e). These temperatures are significantly higher than *T*_c_bullk_ = 1.42 K of SRO in the eutectic crystal used in this study because of enhanced superconductivity at the interface between Ru and SRO, the so-called 3-K phase^22^. A clear supercurrent branch with zero voltage is obvious in an *I*-*V* curve at 0.37 K (inset of Fig. 2a). These facts, as well as Fraunhofer pattern (see the supplementary information), indicate that our junctions exhibit a typical Josephson coupling.

Figure 2a presents *I*_c_ versus temperature data, accumulated from 0.34 K to 2.5 K with various cooling cycles (represented with different colors). Interestingly, we find a sharp jump in *I*_c_ after “each” cooling cycle. Such jumps are prominent at *T* < *T*_c_bullk_; at *T* > *T*_c_bullk_ *I*_c_ is rather stable. The changes in *I*_c_ indicate the switching between two states of the junction with the cooling cycles.

**Figure 3:**
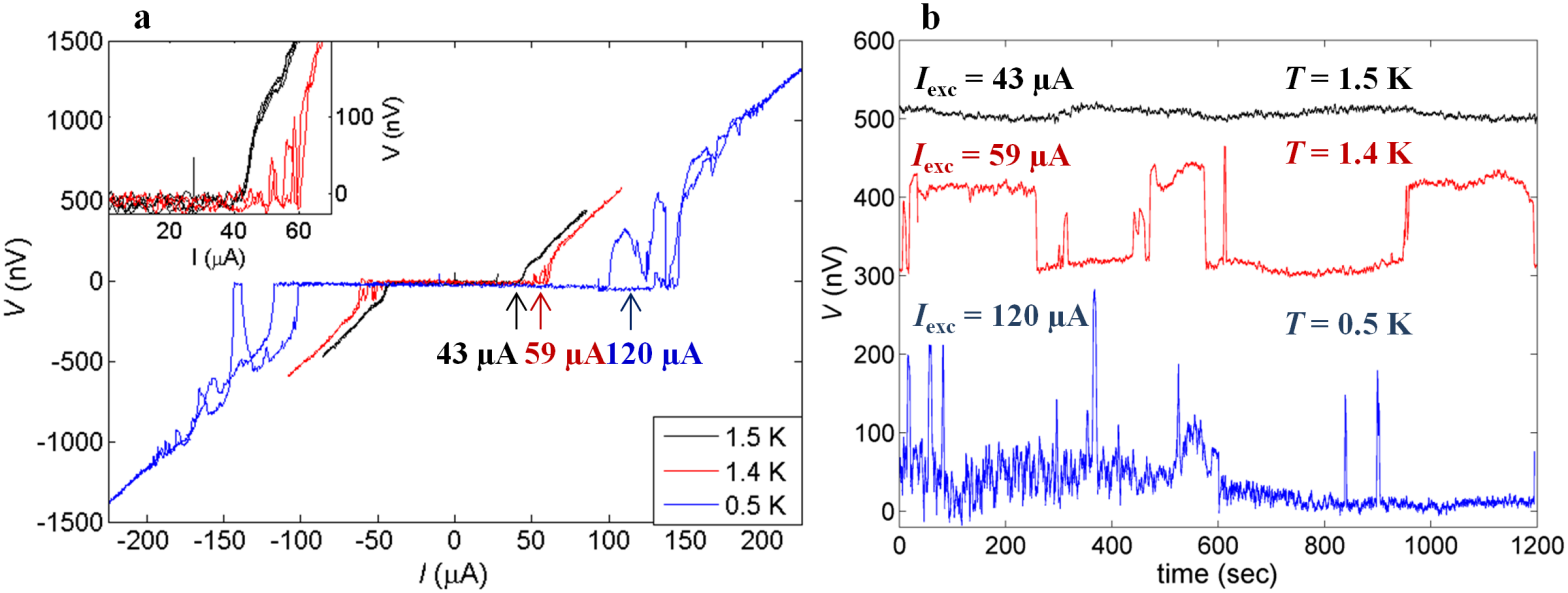
Switching behavior in topological junctions. (a), *I*-*V* curves at various temperatures for Junction B at zero field after zero-field cooling. The *I-V* curve at 1.5 K exhibits ordinary behavior. However, at 1.4 K, and 0.5 K the *I*-*V* curves show switching between zero and non-zero voltages near the transition region. The inset presents the enlarged *I*-*V* curves at 1.5 K and 1.4 K, clearly illustrating that the switching occurs only at 1.4 K. This fact indicates that the switching is strictly connected with the bulk superconductivity of SRO below 1.42 K. (b), Voltage versus time *V*(*t*) with external excitation current *I*_exc_ slightly lower than *I*_c_ recorded just after measuring the corresponding *I*-*V* curves given in the panel (a). At 1.5 K, *V*(*t*) (black; voltage is shifted by 500 nV) is constant with the small extrinsic voltage drift of about 10 nV over a period of 10^3^ sec. At 1.4 K, *V*(*t*) (red; voltage shifted by 300 nV) shows the sharp switching between the lower-*I*_c_ and higher-*I*_c_ states. At lower temperature of 0.5 K, *V*(*t*) (blue) exhibits similar switching but the junction comes back to the higher-*I*_c_ state after a very short time.

To study the *I*_c_ variations further, we obtained *I*-*V* curves at various temperatures after zero-field cooling (Fig. 3a). An ordinary *I*-*V* curve is observed at 1.5 K. However, *I*-*V* curves at 1.4 K and 0.5 K exhibit oscillations between zero and non-zero voltages corresponding to switching between higher-*I*_c_ and lower-*I*_c_ states. At these three temperatures the voltage versus time *V*(*t*) is also recorded at constant excitation current *I*_exc_ just below *I*_c_ (Fig. 3b). At 1.5 K, *V*(*t*) exhibits constant zero voltage. However, at 1.4 K, *V*(*t*) shows sharp switching between zero and non-zero voltages ≈120 nV. This switching resembles telegraphic noise (TN). Nearly equal probabilities in the non-zero and zero voltage states indicate that the lower-*I*_c_ state is as stable as the higher-*I*_c_ state. The *V*(*t*) data at 0.5 K demonstrate rather sharp and short switching at the amplitude of ~200 nV. Thus, the junction tends to stay in the higher-*I*_c_ state. These observations, as well as sudden disappearance of TN signal at *T*_c_bulk_ in data (see the supplementary information) taken under a temperature up-sweep, reveal that the switching behavior is strictly correlated with the bulk superconductivity in SRO; the junction is quite stable at *T* > *T*_c_bullk_ and rather unstable at *T* < *T*_c_bullk_. Note that the switching is also observed at different temperatures between 1.4 K and 0.5 K. Although the transition temperature of bulk Ru is 0.49 K, we do not observe any anomaly in *I*_c_(*T*) at a corresponding temperature (see fig. 2); this observation indicates that Ru is already fully proximitized.

**Figure 4:**
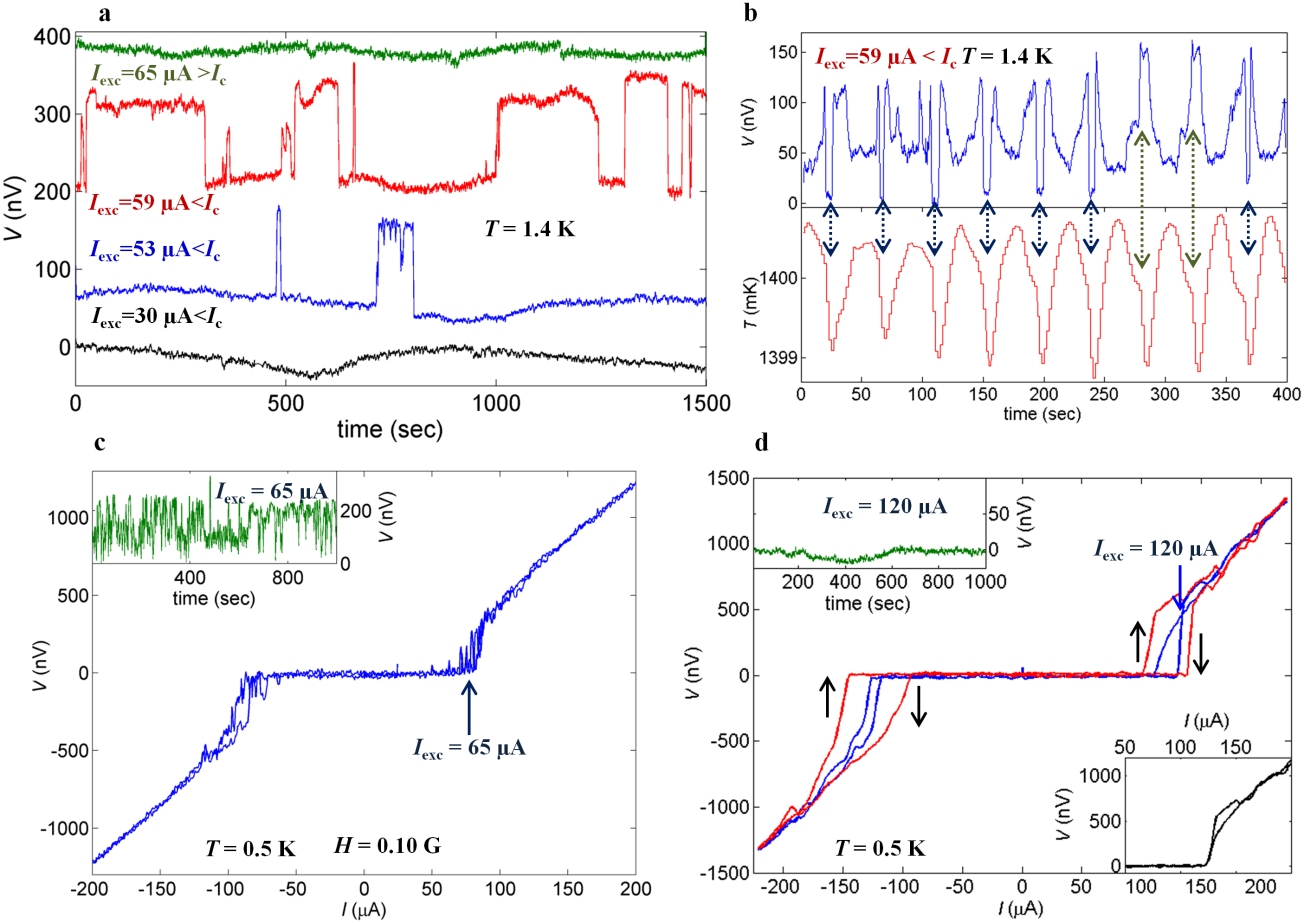
Influence of different external parameters on switching. (a), *V*(*t*) at 1.4 K measured at different *I*_exc_ values. At *I*_exc_ = 30 μA < *I*_c_ ≈ 62 μA, *V*(*t*) (black) represents the zero voltage state. At *I*_exc_ = 53 μA (blue; shift of 70 nV for clarity) switching between lower and higher-*I*_c_ states occurs. At *I*_exc_ = 59 μA close to *I*_c_ (red; shift of 200 nV), the switching occurs more frequently and the junction tends to spend more time in the lower-*I*_c_ state. At *I*_exc_ = 65 μA > *I*_c_ (green: shift of 80 nV) the voltage is constant at ≈300 nV. (b), *I*_c_ switching triggered by temperature variations (≈1.5 mK). *V*(*t*) at 1.4 K shows in-phase voltage oscillations with temperature variations (blue arrows). Note that transition to the lower voltage state is sometimes absent (green arrows). It suggests that temperature variations can control the voltage oscillations but is not the origin. (c), External magnetic-field effect on switching. The *I*-*V* curve was obtained under a field of 0.10 Oe at 0.5 K after zero-field cooling. Although *I*_c_ is suppressed, switching still exists with smaller period of time. *V*(*t*) in the inset also indicates fast switching. (d), Cooling cycle effect on switching at 0.5 K. *I*-*V* curves were obtained in zero field after zero-field cooling. It shows anomalous hysteresis without switching, in contrast to the case shown in Fig. 3a recorded in different cooling cycle. The switching in *V*(*t*) is also absent (upper-left inset), when only the hysteresis is present. The hysteresis can be sometimes absent (lower-right inset).

We also preformed experiments to control the switching behavior. Figure 4a shows the influence of *I*_exc_ at 1.4 K. The *V*(*t*) data at *I*_exc_ = 30 μA, about half of *I*_c_ = 62 μA, show zero resistance. At *I*_exc_ = 53 μA, *V*(*t*) exhibits voltage switching of the order of 120 nV. Note that switching between the high voltage state and an intermediate state is sometimes observed. Overall, a longer time in the zero-voltage state indicates that the junction is more stable in the higher-*I*_c_ state. Closer to *I*_c_ (*I*_exc_ = 59 μA), the junction spends nearly equal time in both states. For *I*_exc_ = 53 μA > *I*_c_, *V*(*t*) exhibits constant non-zero voltage. Thus, the switching is only observed in the *I*_exc_ range where the voltage oscillations are present in the corresponding *I*-*V* curves (Fig. 3a). Close to *I*_c_, where the junctions are rather unstable, we found that a small temperature variation can trigger the switching: in the *V*(*t*) curve at 1.4 K with intentional temperature variations of ~1.5 mK, the switching occurs in-phase to the temperature variations (Fig. 4b). Note that the temperature variations during *V*(*t*) measurements of the curves in Figs. 3&4a were smaller than 50 μK; this fact evidences that the temperature variations can stimulate the switching but is not the origin. We also found that switching is enhanced by small externally applied magnetic fields. Figure 4c shows the *I*-*V* curve at 0.5 K with the field of 0.10 Oe along the *ab*-plane exhibiting fine voltage variations, with the corresponding *V*(*t*) data also showing fast switching (inset of Fig. 4c).

We also demonstrate that switching behavior can be altered by cooling cycles. Figure 3 and Fig. 4d show data at different cooling cycles at 0.5 K. The switching, which is obvious in the former case, is not observed in the latter case. The switching is not observed for *I*_exc_ < *I*_c_ either (upper-left inset of Fig. 4d). It is interesting that the hysteresis loop in *I*-*V* curves for the latter case is reversed, in the sense that zero voltage state is realized with higher current for down sweep. This hysteretic behavior is also anomalous because the difference between the higher and lower *I*_c_ is not constant; sometimes the hysteresis in *I*_c_ disappears (bottom-right inset of Fig. 4d). These facts reveal that the system in this cooling cycle is rather stable but anomalous hysteresis suggests that some instability is present at *I*_exc_ > *I*_c_. Indeed, we observed a small switching only at *I*_exc_ = 144 μA (*I*_exc_ > *I*_c_), but surprisingly not for *I*_exc_ = 143 or 145 μA (see the supplementary information).

## Discussion

Prior to discussion, we summarize the behavior of the Nb/Ru/SRO junctions. With decreasing temperature below *T*_c_Nb_ = 9.5 K, the proximity effect of the *s*-wave superconductivity develops in Ru. Below 3 K, the interfacial 3-K superconductivity in SRO sets in and the junctions start to show finite *I*_c_ below ~1.8 K, forming SNS′ junctions. Although the junction behavior is conventional and highly reproducible down to *T*_c_bullk_, a number of anomalous behaviors emerge at temperatures precisely below *T*_c_bullk_. First is the anomalous hysteresis in the *I*-*V* curves, often accompanied by asymmetry with respect to the direction of current. A similar hysteresis has been reported by Kambara *et al*., in SRO-Ru micro-bridge^18^. Second is the presence of mainly two branches of *I*_c_, between which junctions switch back and forth. Third is the TN, which corresponds to the telegraphic switching between the multiple branches of *I*_c_. The junctions at temperatures just below *T*_c_bulk_ show rather active TN with mainly two different states. At low temperature the junctions are more stable in the higher-*I*_c_ state. Whenever the TN is active *I*_c_ drops down by ≈50% to *I*_c _in the most stable state. The junctions can be driven into unstable state with active TN either by different cooling cycle or by tiny external magnetic fields. Comparing Junctions A and B, Junction A with smaller junction area is relatively stable. These behaviors cannot be explained by the motion of ordinary vortex (see the supplementary information). Below, we examine the possible origins of the unusual behavior in terms of self-induced vortex dynamics specific to chiral superconductor, and in terms of chiral-DW dynamics.

Self-induced vortex: For a Ru-inclusion below its *T*_c_ (0.49 K) surrounded by SRO, it is theoretically predicted that a self-induced vortex appears due to a competition between *s*-wave superconductivity in Ru metal and the chiral *p*-wave superconductivity in SRO. Such a vortex can switch between two states: one at the Ru/SRO interface and the other at the center of the Ru-inclusion. The switching should occur at lower temperatures and more likely for smaller Ru-inclusions^23^. In our junctions, similar self-induced vortex is anticipated even above 0.5 K because of the proximity-induced superconductivity in Ru via Nb electrode. However, our junctions become stable at lower temperatures and also for smaller Ru-inclusion. Thus, the observed behavior is unlikely caused by the self-induced vortex dynamics.

**Figure 5:**
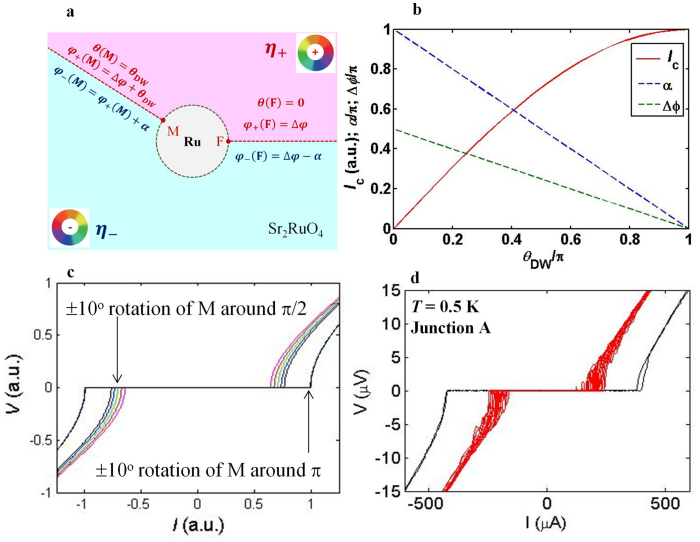
*I*_c_ switching originating from chiral domain wall motion. (a), Schematic of Sr_2_RuO_4_ sample with one Ru inclusion. The figure represents the NS′ part of an SNS′ (Nb/Ru/Sr_2_RuO_4_) junction, where N (Ru-metal) is proximitized by conventional *s*-wave superconductor S (Nb). Two chiral domain walls intersecting the interface between Ru and SRO are also shown. One domain wall “F” is considered to be fixed at angle *θ* = 0 with the phase difference between the *s*-wave and the *p*-wave superconductors *φ*_+_(F) = Δ*φ*. The second domain wall “M” is considered to be movable and located at *θ* = *θ*_DW_ with *φ*_+_(M) = *θ*_DW_ + Δ*φ*. For the maximum critical current for a given value of *θ*_DW_, phase differences across the domain walls are *α*(M) = −*α*(F) = *π* − *θ*_DW_. The color wheels represent the evolution of the superconducting phase *φ* with the direction *θ*. (b), Calculated *I*_c_, *α*, and Δ*φ* as functions of *θ*_DW_. Maximum *I*_c_ is realized at *θ*_DW_ = *π* with *α* = Δ*φ* = 0. (c), Calculated *I*-*V* curves for domain wall motion over ±10° around *θ*_DW_ = *π* and *θ*_DW_ = *π*/2. (d), Experimentally obtained *I*-*V* curves at 0.5 K in two different cooling cycles. Red curves are representing many traces of current sweep without changing the temperature.

Chiral-DW dynamics: Chiral domain structure of superconducting bulk SRO around the Ru-inclusion is expected to play a crucial role in determining *I*_c_. It is considered to appear only below *T*_c_bullk_^20^,^21^, in accord with the emergence of unusual behavior observed only below *T*_c_bullk_. Expectedly, it becomes more stable at low temperatures with increasing condensation energy of SRO. To illustrate the effect of chiral-DW dynamics, let us introduce a simplest model. We consider a single Ru-inclusion having a smooth circular-shape in pure SRO and two chiral-DWs separating the imbedded in SRO into two domains with opposite chirality (*η*_+_ = *e^iθ^* and *η*_−_ = *e*^−*iθ*^, Fig. 5a). The chiral-DWs intersect with the Ru/SRO interface fixed at *θ*(F) = 0° with *φ*_+_(F) = Δ*φ* (*θ* is the direction normal to the interface, *φ*_±_ is the phase difference of the *p*-wave SRO in the domain *η*_±_ with respect to the *s*-wave Ru) and at *θ*(M) = *θ*_DW_ which is assumed movable. The free energy of a chiral-DW depends on the orientation and the phase difference *α* across the chiral-DW, which would be determined by minimizing the junction energy for a given “M” and *I*_exc_ with the distribution of the pinning potential for chiral-DWs, etc. in an intricate way^16^. Here, the effect of the additional energy associated with the induced magnetic flux is not included. This is because the phase winding mismatch between s-wave and p-wave is resolved by presence of a chiral domain wall and as a consequence the flux energy is expected to be much reduced. Thus, we introduce a conjecture that *I*_c_ is determined by the following current-phase relation for a given *θ*_DW_ which is varied from 0 to *π*, 
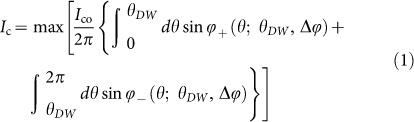
By symmetry and for single-valuedness of the order parameter we consider *α*(M) = −*α*(F) = *π* − *θ*_DW_. This in fact is the condition to maximize *I*_c_ for a given value of *θ*_DW_. In this scenario, maximum *I*_c_ is realized at *θ*_DW_ = *π* and *α* = Δ*φ* = 0 (Fig. 5b). This can be understood as the absence of current cancelation over the Ru circumference in the presence of multiple chiral-DWs (see the supplementary information). A rotation of domain wall M by ±10° around *θ*_DW_ = *π* affects *I*_c_ very little, but the rotation around *θ*_DW_ = *π*/2 significantly changes *I*_c_ (Fig. 5c; *I*-*V* curves are calculated using the relation for an overdamped junction). This character captures features of our experimental observations (Fig. 5d): stable and maximum *I*_c_ is observed in one cooling cycle, and unstable and lower *I*_c_ is realized in a different cooling cycle. In reality, it is reasonable that *θ*_DW_ = *π* gives the most stable state because the actual Ru inclusion is elongated with the maximum curvature (maximum disorder) at its corners providing maximum pinning for chiral-DWs. Note that the observed *I*_c_ is always ~50% lower when voltage oscillations emerge in the *I*-*V* curves. In our model such lower and unstable *I*_c_ occurs for the chiral-DW motion around *θ*_DW_ = *π*/2, corresponding to the flat part of the actual Ru/SRO interface. These calculations confirm that the aspects of the observed anomalous behavior of our junctions are well explained by the chiral-DW motion.

We studied Sr_2_RuO_4_-based micron-sized junctions, Nb/Ru/SRO, using one Ru inclusion, and found unusual temperature dependence of *I*_c_, anomalous hysteresis with current, and switching in *I*_c_. It is difficult to explain the overall behavior by vortex dynamics (ordinary and self-induced). Instead, a simple model based on the chiral-DW motion captures the main features of the observed junction behavior. Our results provide further evidence for chiral *p*-wave order parameter in SRO and reveal the crucial effects of chiral-DW motion on Josephson coupling. The switching raised by chiral-DW motion can be controlled by various external parameters and provides a ground for novel superconducting devices, analogous to memory devices based on ferromagnetic-DW motion. Our work also demonstrates the scientific importance of the concept of the topological junctions to expose the phase winding of superconducting order parameter by making use of the real-space topology.

## Methods

We fabricated Nb/Ru/SRO micron-sized Josephson junctions using a polished (the basal *ab*-plane) rectangular pieces (3 × 3 × 0.5 mm^3^) of SRO-Ru eutectic crystals grown by a floating-zone method^24^. Contact resistance between the *ab*-surface of SRO and Nb is rather large but Ru metal works as an adhesive layer to provide a good contact. Although, a technique of using Nb/Cu bilayer has recently been developed to establish a good contact to the surface parallel to the *c*-axis to enhance the *J*_c_ of the junction^25^, here we need to deal with the *ab*-plane surface contact. After polishing its *ab*-surface, SiO_x_ layer of thickness of ~300 nm was deposited using RF sputtering technique with a backing pressure of ~10^−7^ mbar. Then a photoresist (TSMR-8800) was coated, exposed with laser lithography over only one Ru inclusion. The exposed resist was removed with TMAH2.83% developer for 120 sec followed by rinsing in DI-water for 30 sec and dried with N_2_ gas. A part of the SiO_x_ film covering single Ru inclusion was etched with CHF_3_ gas, which opened the windows over a single Ru inclusion (Fig. 1a). In this process, a fluoride thin film may be generated on the surface of the sample. We performed an O_2_ plasma cleaning step to etch away a fluoride film. The resist was removed using N-Methyl-2-pyrolidone (NMP) and cleaned with acetone and isopropanol. In the next step to deposit the Nb electrodes, we used a lift-off technique using bilayer photoresist (LOR-10A and TSMR-8800) and laser lithography. A Nb film of the thickness of ~1 μm was sputtered with a base pressure of ~10^−7^ mbar. Finally, the lift-off was accomplished with NMP. Note that Nb is not only in contact with Ru but also with SRO along *ab*-plane. It is well known that *ab*-plane is less conductive because of atomic reconstruction at the surface and does not allow supercurrent to flow directly from Nb. Instead, supercurrent from Nb passes only through the Ru metal with proximity induced superconductivity from Nb. Figure 1b shows an overall scanning electron microscope (SEM) picture of the device with two junctions. We measured the *I*-*V* curves using four-point technique with two contacts at Nb over the *ab*-plane and the other two contacts on the side directly connected via silver paste with SRO crystal as shown in the schematic of the side view in Fig. 1c. The measurements are performed with a ^3^He cryostat down to 300 mK. The cryostat was magnetically shielded with high-permeability material (Hamamatsu Photonics, mu-metal). Inside the shield, we placed a superconducting magnet to apply the magnetic fields.

## Supplementary Material

Supplementary Information Supplementary Informations
