# Supplementary material for: Anomalous switching in Nb/Ru/Sr2RuO4 topological junctions by chiral domain wall motion
Source: Sci Rep. 2013 Aug 21;3:2480. doi: 10.1038/srep02480 (PMC6505398; doi:10.1038/srep02480)
Supplement: Supplementary Information — s [file srep02480-s1.doc]

**Supplementary information for**

**Anomalous awitching in Nb/Ru/Sr_2_RuO_4_ topological junctions by chiral domain wall motion**

M. S. Anwar^1^, Taketomo Nakamura^1,2^, S. Yonezawa^1^, M. Yakabe^3^,

R. Ishiguro^3^, H. Takayanagi^3,4^ and Y. Maeno^1^

^1^Department of Physics, Kyoto University, Kyoto 606-8502, Japan

^2^Institute for Solid State Physics, the University of Tokyo, Kashiwa277-8581, Japan

^3^ Department of Applied Physics, Faculty of Science, Tokyo University of Science, Tokyo 162-8601, Japan

^4^International Center for Materials Nanoarchitectonics (MANA), National Institute for Materials Science (NIMS), Tsukuba 305-0044, Japan

This supplementary information provides additional data and discussion to elaborate the key results given in the main text. Here, Fraunhofer pattern, asymmetric *I*-*V* curves, voltage oscillations (telegraphic noise: TN) as a function of temperature, TN at 0.5 K for *I*_exc_ > *I*_c_, and the effect of temperature variations on voltage oscillations are presented. We also discuss further details of the origin of the switching behavior.

Before going into details, we would like to describe characteristic times involved in data acquisition. These characteristic times are important to understand the data, in particular the telegraphic noise. The filtering time and the sampling time of the data acquisition are both much shorter than the transition time, duration, and the frequency of a telegraphic event. We used a Nanovoltmeter Keithley-2182 with the medium speed setting (the filtering time of 50 msec) and set the sampling time of 50 msec. With these settings we observed the transition time of a telegraphic event of the order of 2.5 sec at 1.4 K and 1.8 sec at 0.5 K, which is two orders of magnitude longer than the time of filtering and sampling. The duration of a telegraphic event is of the order of 6.7 sec at 1.4 K and 2.27 sec at 0.5 K. Thus, it is unlikely that we miss a telegraphic event. We cannot be sure that we do not miss telegraphic events above the bulk *T*_c_, but it requires that the characteristic times of an event becomes three orders of magnitude shorter above the bulk *T*_c_.

SRO is a good metal and has the bulk superconducting transition around 1.42 K. We observe superconducting critical current density *J*_c_ above *T*c_bulk because of 3-K phase (interfacial superconductivity induced at the interface between Ru and SRO). Very low resistivity of bulk SRO (~ 0.1 µΩcm) gives the normal resistance of the order of µΩ just before the *T*c_bulk. It reveals that below junction *J*_c_ may well contain the normal resistance of the bulk path (~ 0.2 µΩ), which is negligible compared to the junction normal resistance (≈ 7 mΩ for Junction B and 27 mΩ for junction A). What is certain from the data is that below 1.86 K the junction area establishes the SNS’ junction with the development of superconductivity at the interface between Ru and SRO that defines the *J*_c_ of the junction. That is what revealed by the AC and DC susceptibility measurements, the volume fraction near the *T*c_bulk is corresponding to the bulk value [1].

**Fraunhofer pattern**

SNS’ junctions made of Nb/Ru/Sr_2_RuO_4_ (SRO) exhibit usual behavior of critical current *I*_c_ as a function of applied magnetic field termed as Fraunhofer pattern as shown in Fig. S1. The Fraunhofer pattern for Junction B at 0.5 K with applied field along the *ab*-plane shows clear minima at ±130 Oe and some additional shoulders at lower fields. The junction area corresponding to ±130 Oe is ≈ 0.16 µm^2^. However, the geometry of our topological junction is rather complicated to calculate the junction area which contributes to the *I*_c_ modulation. At 0.5 K the proximity penetration depth in the Ru inclusion is of the order of 1 µm. By considering 1 µm long interface between SRO and Ru, which is the main junction, yields the junction depth of the order 160 nm inside the SRO. The additional shoulders suggest mechanism related to dynamical nature of the junction.

**Temperature dependent voltage oscillations**

Our junctions exhibit stable *I*_c_ down to the bulk superconducting transition temperature of SRO (*T*c_bulk) but a number of anomalous features at lower temperatures. Figure S2 shows voltage variations at constant excitation current (*I*_exc_) as a function of time for Junction A (upper panel); the temperature is gradually increased with time (lower panel). The large voltage variations stop at *T* = 1.423 K, which corresponds to *T*c_bulk of SRO. This fact illustrates that the anomalous behavior emerges from the chiral superconductivity of SRO and is most probably connected with dynamic nature of its chiral domain structure.

**Asymmetric *I*-*V* curves**

The observed *I*-*V* curves often become asymmetric with the direction of the current (difference between *I*_c+_ and *I*_c-_) for *T* < *T*c_bulk (Fig. S3) but are always symmetric for *T* < *T*c_bulk. It is consistent with previous observations Nakamura *et al.,*^2,3^. Note that the sign and magnitude of the observed asymmetry varies with cooling cycles. Asymmetric *I*-*V* curves are observed for a SQUID consisting on two non-identical junctions. In case of Josephson junction asymmetric *I*-*V* curves may result from non-identical interfaces. But in both of these cases the asymmetry is persistent with thermal cycles^4^. In such a case, the asymmetry is intrinsic to each junction device and persists in every cooling cycle, in contrast with the present observations. This fact suggests that our junctions have different chiral-DW states during different current sweeps.

**Telegraphic noise at *I*_exc_ > *I*_c_ for relatively stable state**

The TN is mainly observed for *I*_exc_ lower than but close to *I*_c_. Sometimes TN is also observed for *I*_exc_ > *I*_c_. As an example at 0.5 K we present TN at
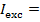
 144 µA
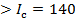
 µA in Fig. S4. It is interesting to note that TN is very sensitive to
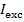
: if we increase or decrease *I*_exc_ just by 1 µA the TN disappears. Voltage is time independent at *I*_exc_ = 143 µA and 145 µA but the voltage value is larger for smaller *I*_exc_, exhibiting non-ohmic behavior. This observation suggests that the chiral-DW motion may affect the junction resistance in resistive regime beyond *I*_c_.

In this situation the reverse hysteresis is also observed. The *I*_c_*R*_N_ values for our junctions is in the range of 5-15 µV, which is much smaller than the superconducting gap. It reveals that our junctions are underdamped (very small McCumber parameter) and non-hysteretic *IV*s are expected for conventional junctions. Thus, inverse hysteresis is not coming from the quality of the junction; it might also be related with other mechanisms such as the chiral DW dynamics.

**Influence of temperature variations on the switching**

In the main text we presented the *V*(*t*) data at 1.4 K (Fig. 4b) for which TN are controlled by temperature variations even of the order of 1.5 mK. But at 0.5 K the *V*(*t*) data do not show TN in many cases (Fig. 4d). The *I*-*V* curve in the former case also exhibit voltage oscillations with respect to *I*_exc_ but not in the latter case. When *I*-*V* curves do not show voltage oscillations the junction is in a stable and higher-*I*_c_ state (e.g. Fig. S3 and Fig. 4d). In this case, the system is found to be rather stable even with larger temperature variations. The *V*(*t*) data at 0.5 K in Fig. S5a present the stable state with temperature variations of the order of 50 µK. The *V*(*t*) data exhibit essentially the same constant voltage even with temperature variations of the order of 4 mK (Fig. S5b).

**Possible origin of the switching - ordinary vortex motion**

The ordinary vortex dynamics can cause TN with the motions of the vortices from the Ru/SRO interface to the bulk SRO, or vice versa. In this context the TN may be explained with pinning and depinning of the vortices, but it is difficult to explain the anomalous hysteresis. Furthermore, the effect of ordinary vortices, if playing a main role, should be initiated with the onset of the 3-K superconductivity or at least at 1.8 K where
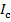
 emerges. In contrast, the observed TN is strictly connected with the bulk superconductivity in SRO. In addition, we emphasize that we tried to minimize the effect of trapped vortices by cooling the junctions rather slowly (1 K/hour) in a zero field environment prepared by magnetic shielding.

**Relation between the real-space angle and superconducting phase**

We also confirmed analytically that maximum *I*_c_­ for given *θ*_DW_ is realized at *α*(M) = -*α*(F), which is the most stable domain walls configuration. Figure S6 represents the superconducting phase *ϕ* of Sr_2_RuO_4_ and local critical current *I*_co_sin*ϕ* as a function real-space angle *θ* around Ru inclusion for two different positions of chiral-DW “M” at *θ*_DW_ *=* π/2 and *θ*_DW_ *=* π, calculated based on the model described in the main text. For *θ*_DW_ *=* π/2, negative local *I*_c_ in certain ranges of *θ* reduces the total *I*_c_, which is given by the integration around the Ru/SRO circumference. Maximum total *I*_c_ is realized for *θ*_DW_ *=* π, forwhich the negative contribution is absent.

**Figure S1| Critical current versus external applied field.** Fraunhofer pattern with field applied along the *ab*-plane for Junction B at 0.5 K. It shows a clear dips at 130 Oe with additional shoulders at lower field.


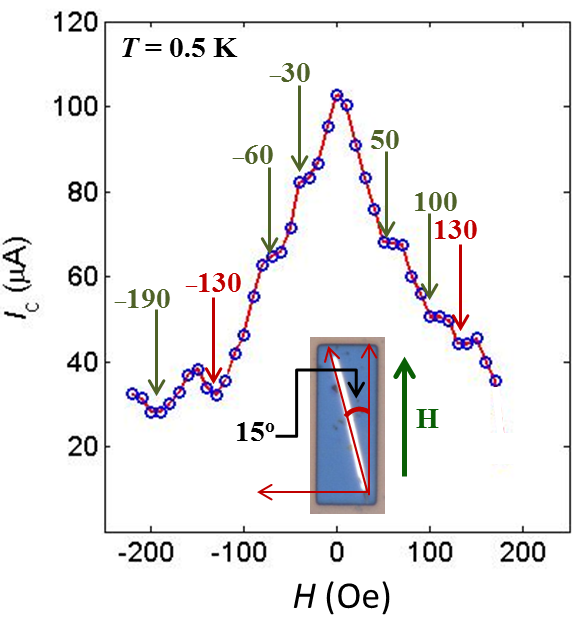


**Figure S2| Temperature dependence of voltage oscillations.** Voltage oscillations versus temperature for Junction A. Upper panel shows the switching of voltage in higher and lower voltage states and lower panel illustrates the increase in temperature as a function of time. The oscillations are totally suppressed just at 1.423K (*T*c_bulk of SRO).


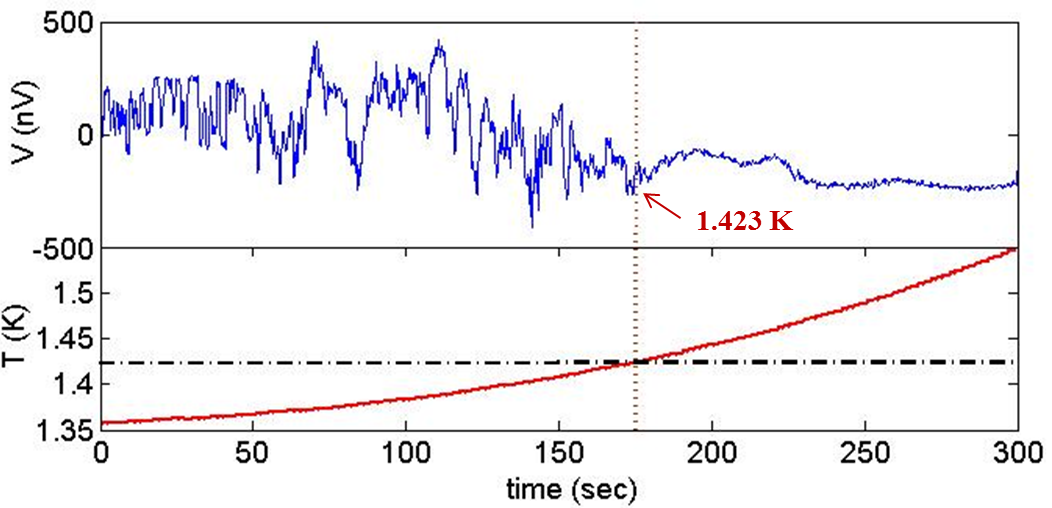


**Figure S3| Asymmetric *I*-*V* curves.** Asymmetric *I*-*V* curves with the direction of the current at different temperatures for Junction A. The difference between *I*_c_+ and *I*_c_- is obvious with
 30 µA(blue), 27 µA(red), 75 µA(black). During other thermal cycles, we also observed the telegraphic noise in this temperature range.


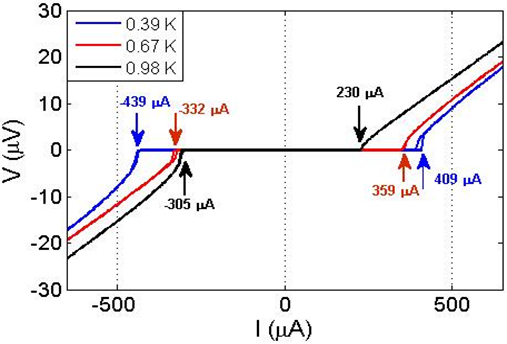


**Figure S4| Switching at
 and 0.5 K.** Telegraphic noise for Junction B at different *I*_exc_ values. Telegraphic noise is present at *I*_exc_ = 144 µA with the voltage amplitude of ~60 nV. But the voltage turns to be constant just with a change of ±1 µA in *I*_exc_ (145 µA and 143 µA). Note that the junction voltage at 145 µA is lower than that of at 143 µA. It opposes the Ohmic behavior. This behavior implies a subtle instability to govern the critical current in this junction.


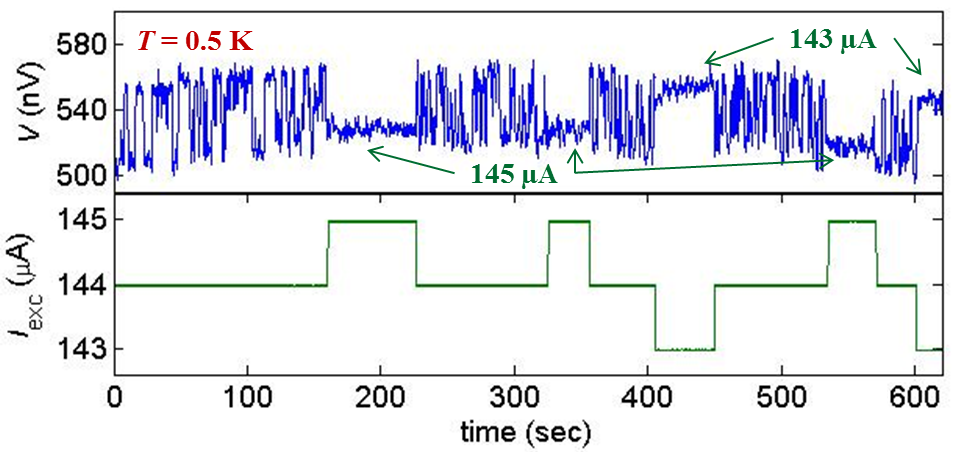


**Figure S5| Voltage switching dependence on temperature variations.** **a**, Voltage versus time at 0.5 K for Junction A. It illustrates zero voltage without any switching with very stable temperature. **b**, Voltage versus time with much larger temperature variations. It shows again the constant voltage although the temperature variations are of the order of 4 mK.


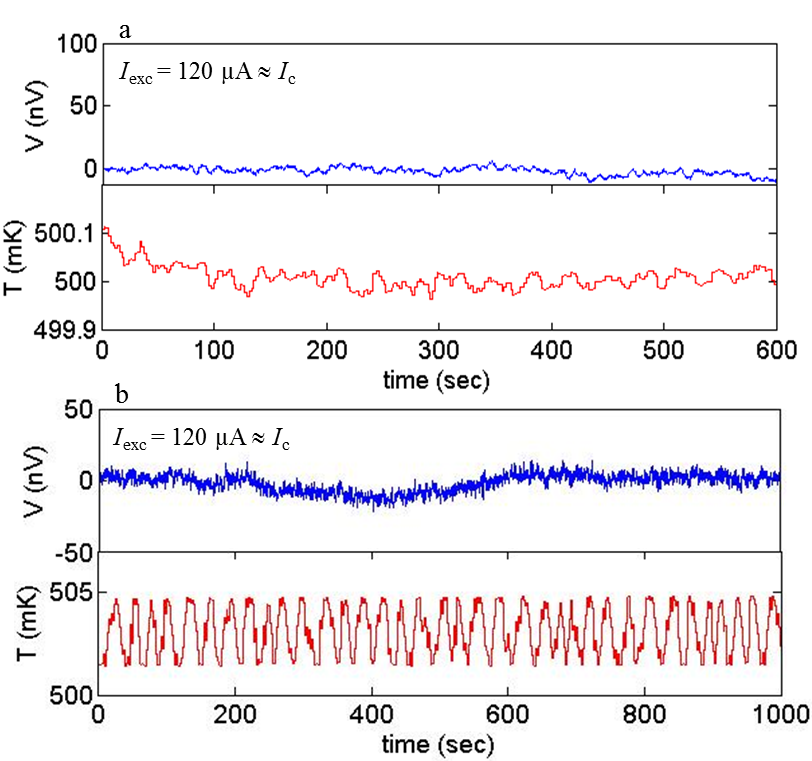


**
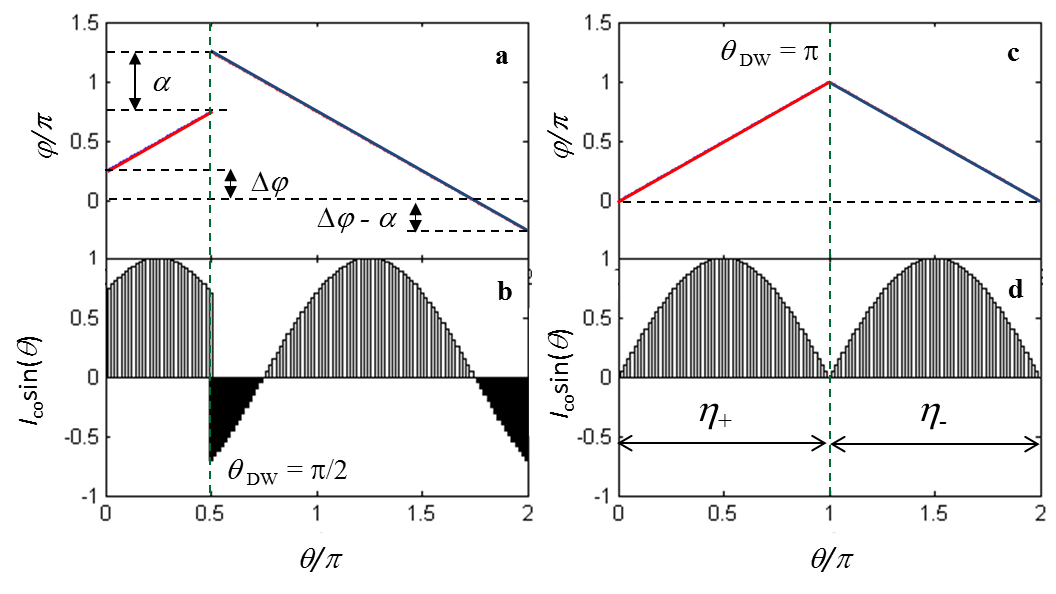
**

**Figure S6| Superconducting phase and local critical current versus position around Ru inclusion. a,** Superconducting phase *ϕ* of chiral *p*-wave superconductivity for *θ*_DW_ *=* π/2 with the phase jump *α* across the chiral-DW. The red and blue lines represent the phase in the positive and negative chiral domains, respectively and vertical green dotted line represents the value of *θ*_DW_. **b,** Local critical current versus angle *θ* for *θ*_DW_ *=* π/2. Positive and negative local *I*_c_ is presented by gray area and black area, respectively. **c,** Superconducting phase *ϕ* as a function of *θ* for *θ*_DW_ *=* π and α = 0. **d,** Local critical current versus angle *θ* for *θ*_DW_ *=* π. Maximum total *I*_c_ is realized for this configuration.

**References**

1. Kittaka, S., Taniguchi, H., Yonezawa, S., Yaguchi, H. and Maeno, Y., Higher-Tc superconducting phase in Sr2RuO4 induced by uniaxial pressure, *Phys. Rev. B* **81,** 180501(R) (2010).
2. Nakamura, T., Nakagawa, R., Yamagishi, T., Terashima, T., Yonezawa, S., Sigrist, M., & Maeno, Y., Topological competition of superconductivity in Pb/Ru/ Sr_2_RuO_4_ junctions. *Phys. Rev. B* **84**, 060512 (2011).
3. Nakamura, T., Sumi, T., Yonezawa, S., Terashima, T., Sigrist, M., Kaneysasu, H., & Maeno, Y., Essential configuration of Pb/Ru/ Sr_2_RuO_4_ junctions exhibiting anomalous superconducting interference. *J. Phys. Soc. Jpn* **81,** 064708 (2012).
4. Nelson, K. D., Mao, Z. Q., Maeno, Y., & Liu, Y., Odd-parity superconductivity in Sr_2_RuO_4_. *Science* **306**, 1151–1154 (2004).
